# Fatal bacteremia caused by *Staphylococcus argenteus*: A case report

**DOI:** 10.1097/MD.0000000000035866

**Published:** 2023-11-17

**Authors:** Yu Zhan, Ruizhi Tang, Yingmiao Zhang, Xun Li, Yue Fan, Jing Wang, Hui Wang, Zhongxin Lu

**Affiliations:** a Department of Medical Laboratory, The Central Hospital of Wuhan, Tongji Medical College, Huazhong University of Science and Technology, Wuhan, China; b Cancer Research Institute of Wuhan, The Central Hospital of Wuhan, Tongji Medical College, Huazhong University of Science and Technology, Wuhan, China.

**Keywords:** 16S rRNA, bacteremia, case report, MALDI-TOF MS, *Staphylococcus argenteus*

## Abstract

**Rationale:**

*Staphylococcus argenteus (S argenteus*) is a novel and emerging species that is part of the *Staphylococcus aureus (S aureus*) complex. Fatal cases of bloodstream infection caused by *S argenteus* are rarely reported and should be considered in medical practice.

**Patient concerns:**

A 44-year-old male was admitted to our hospital with reduced appetite, high fever and unconsciousness. Laboratory tests indicated infection, muscle damage, and alkalosis in the patient. Brain computed tomography (CT) demonstrated small hematoma in left frontal lobe with peripheral cerebral edema. Chest CT demonstrating chronic bronchitis, emphysema, and bullae in the right lung. Blood culture was collected on the first day of hospitalization for microbial culture and pathological examination.

**Diagnosis:**

The isolate from blood culture was identified as *S argenteus* by MALDI-TOF MS after the patient death.

**Interventions:**

The patient was subjected to empirical antibiotic treatment with piperacillin/tazobactam.

**Outcomes:**

After 48 hours of hospitalization, the patient died after ineffective rescue.

**Lessons:**

The patient had long-term heavy drinking and smoking as well as chronic malnutrition, which may account for his immune deficiency. The immunocompromised people are more vulnerable to infection by *S argenteus* and then develop bacteremia. The use of piperacillin/tazobactam may have contributed to the patient death.

## 1. Introduction

*Staphylococcus argenteus*, a divergent staphylococcal species that is closely related to *Staphylococcus aureus* and considered as part of the *S aureus* complex, was first described in 2006.^[1,2]^ *S argenteus* may have been overlooked due to limitations of routine methods for clinically microbiological identification. *S argenteus* was initially isolated in aboriginal communities in Northern Australia.^[3]^ Subsequently, several studies reported sporadic cases of infections caused by *S argenteus* in Asia, Oceania, the Pacific Islands, Latin America, Europe, and Africa.^[3–6]^ Here, we describe a fatal case of bacteremia caused by *S argenteus* isolated from bloodstream of a comatose patient.

## 2. Case presentation

On January 1, 2022, a 44-year-old male with obstructive pulmonary disease and renal insufficiency was brought into our emergency department by his family due to reduced appetite, high fever and unconsciousness. According to his family, he had weight loss in recent half a year and a history of heavy drinking and smoking (12 cigarettes/d). His family also reported that he had no history of hypertension, diabetes, and drug abuse, neither history of Covid-19 4 weeks prior to his admission.

Physical examination revealed a comatose male, with a blood pressure of 115/56 mm Hg, body temperature of 39.5°C, respiratory rate of 26 breaths/min, a heart rate of 110 breaths/min, and blood oxygen saturation of 95%. Laboratory tests revealed the following: C-reactive protein of 3.5 mg/dL, white blood cell count of 13.69 × 10^9^/L (87.4% neutrophils), creatine kinase of 889.1U/L, lactate dehydrogenase of 667 U/L, α-hydroxybutyrate dehydrogenase of 509 U/L, aspartate aminotransferase of 195.2 U/L, blood PH value of 7.59, total protein of 63.7g/L, and albumin of 26.2g/L. The electrolyte test of blood revealed decreased potassium (3.44mmol/L), sodium (132.4mmol/L), and calcium (0.98mmol/L). These results indicated infection, muscle damage, and alkalosis in the patient. The Toluidine Red Unheated Serum Test was negative and the Treponema pallidum IgG chemiluminescence immunoassay was 18.26 S/CO. The Toluidine Red Unheated Serum Test is used to assess disease activity and chemiluminescence immunoassay is the mainstay of syphilis screening. The results indicated that the patient had syphil in the past and was not currently active. The patient was subjected to empirical antibiotic treatment with piperacillin/tazobactam (2.5g/12h).

**Figure 1.**
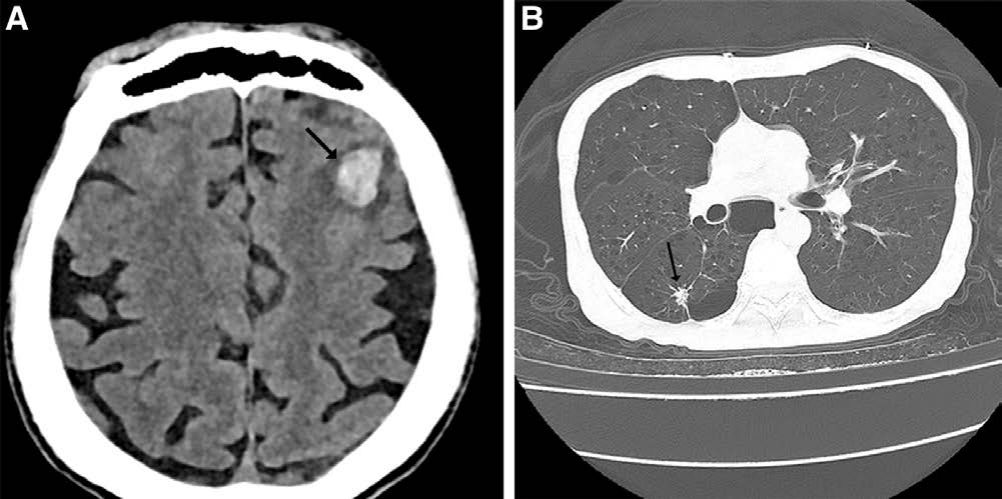
Computed tomography (CT) features in brain and chest. (A) Brain CT demonstrating small hematoma in left frontal lobe with peripheral cerebral edema. (B) Chest CT demonstrating chronic bronchitis, emphysema, and bullae in the right lung.

Emergency brain computed tomography (CT) showed a small hematoma in the left frontal lobe with peripheral cerebral edema, lacunar cerebral infraction, cerebral atrophy, calcification in the left frontal sulcus, a small subarachnoid hemorrhage, and a right sphenoid sinus cyst (Fig. 1A). Chest CT showed chronic bronchitis, emphysema, bullae and multiple old nodules in the right lung. (Fig. 1B). It seems that the dysfunction of brain and lung is not the cause of the patient rapid death.The patient was transferred to intensive care unit after his admission. Two hours later, with Glasgow Coma Score of 5 points, the emergency rescue was applied. Continuous dose of noradrenaline was initiated at a dose of 0.2 μg/kg/min. Bilateral emphysema and right pleural effusion was found by digital radiography on 3 January. After 48 hours of hospitalization, oxygen saturation of the patient was decreased progressively. The patient blood oxygen saturation is difficult to improve after treatment. Finally, the patient died after ineffective rescue. However, blood culture which collected on the first day of hospitalization did not report positive until the patient died. It grew *S argenteus* which was identified by MALDI-TOF MS (Bruker Daltonik GmbH, Germany) with a high confidence level and named *S argenteus* strain X17.

The minimum inhibitory concentration (MIC) of *S argenteus* for antibiotics were determined by Vitek2 (bioMerieux) (Table 1). *S argenteus* is sensitive to levofloxacin, linezolid, oxacillin, erythromycin, clindamycin, gentamicin, ceflorin, rifampicin, moxifloxacin, daptomycin, sulphamethoxazole/trimethoprim, vancomycin, and teicoplanin, but is resistant against benzylpenicillin. The E-test method was performed to obtain the exact MIC value of benzylpenicillin, which was 0.75µg/ml. We detected penicillinase by nitrocefin-based test and found it positive.

**Table 1:** Antimicrobial susceptibility of *S argenteus*.

Antimicrobial agent	MIC(μg/mL)	Interpretation
Teicoplanin	≤0.5	S
Trimethoprim-sulfamethoxazole	≤10	S
Vancomycin	1	S
Rifampicin	≤0.5	S
Levofloxacin	0.5	S
Moxifloxacin	≤0.25	S
Inducible clindamycin resistance		negative
Erythromycin	≤0.25	S
Clindamycin	0.25	S
Linezolid	2	S
Daptomycin	0.25	S
Gentamicin	≤0.5	S
Ceflorin	≤0.06	S
Oxacillin	≤0.25	S
Benzylpenicillin	≥0.5	R

MIC = minimum inhibitory concentration.

**Figure 2.**
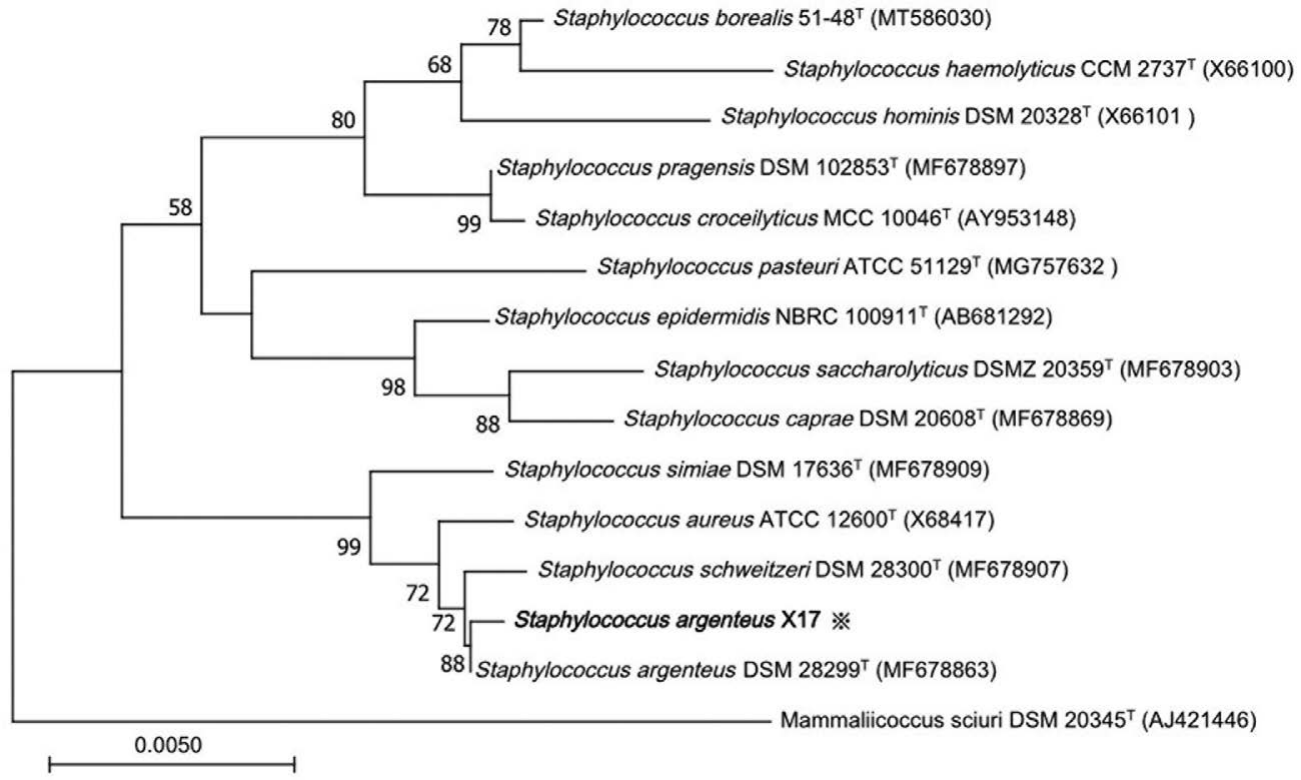
The phylogenetic tree based on the 16S rRNA gene sequences showing the relationship of isolated strain (star) and members within the genus *Staphylococcus*. The tree was reconstructed by the neighbor-joining method, and *Mammaliicoccus sciuri* DSM 20345^T^ was used as an outgroup. Bootstrap values (>50%) based on 1000 replicates are shown at branch nodes. T = type strain.

16S rRNA gene sequence analysis was conducted to classify the isolated strain with universal 16S rRNA primers (forward primer: 5’-AGTTTGATCMTGGCTCAG-3’, reverse primer: 5’-GGTTACCTTGTTACGACTT-3’). A total of 1456 contiguous nucleotides were determined. The complete 16S rRNA sequence was analyzed was with the EZ taxonomy database.^[7]^ The strain X17 exhibited highest (99.18 %) 16S rRNA gene sequence similarity with the type strain of *S argenteus* DSM 28299^T^ (GenBank accession no. MF678863). Multiple alignments with sequences of the most closely related *staphylococci* and the calculations of the levels of sequence similarity were carried out using CLUSTALW.^[8]^ A phylogenetic tree was constructed using the neighbor-joining method by using MEGA software version 11.^[9]^ The topology of the phylogenetic tree was evaluated by using the bootstrap resampling method of Felsenstein^[10]^ with 1000 replicates. The phylogenetic tree showed that the strain was clustered with DSM 28299^T^, and this cluster was strongly supported with a bootstrap value of 88 % (Fig. 2).

## 3. Discussion

Bacteremia is defined as blood stream infection (BSI) that is characterized with the existence of pathogens in the blood. Transient bacteremia is appeared without any symptoms that is limited to 1 or 2 days. Staphylococcal species are usually the causative agent of transient BSI. *Staphylococci* are one of the most common compositions of human microbiota isolated from bloodstream. Staphylococcal infections have different localization, manifestation, and/or courses of disease. The most frequent infections are local infections, which become bacteremia when *staphylococci* invade into the bloodstream and colonize to cause BSI.^[11]^ *Staphylococci* have a great ability to infect human hosts by adherence, invasion, persistence and evasion of the host immunity.^[12]^

S argenteus is a novel and emerging species that is part of the *S aureus* complex. In the past, it was considered as *S aureus* clonal complex CC75.^[1,2]^ It causes infections similar to those triggered by *S aureus*, including skin and soft tissue infections, respiratory infections, necrotizing fasciitis, bone and joint infection, endocarditis, aortic mycotic aneurysm, bacteremia and sepsis, and food poisoning.^[13–16]^ After the first report of genetically divergent ST75 in Australia in 2002, *S argenteus* were increasingly reported globally, including Cambodia, Trinidad and Tobago, New Zealand, Fiji, France, Thailand, China, Belgium, Laos, Japan, Denmark, Myanmar, Sweden, Taiwan, German, Nigeria, Canada and the United States, England, and Brazil.^[13,17,18]^ Chen et al reported that compared with patients with methicillin-susceptible *S aureus* (MSSA) bacteremia, patients with *S argenteus* bacteremia had a higher percentage of polymicrobial blood culture results (*P* = .047), prior healthcare-associated exposure within the past year (*P* = .026), recent hospitalization within 3 months (*P* = .027), thrombocytopenia (*P* = .027), and the respiratory tract as the primary focus of infection (*P* = .028). The patients with *S argenteus* bacteremia had a significantly lower chance of survival than patients with MSSA bacteremia (*P* = .015).^[13]^ Chantratita et al showed that compared with individuals with *S aureus* infection, the rates of bacteremia in patients with *S argenteus* infection were comparable between the 2 groups (32.8% versus 39.5%, adjusted OR 0.65, 95% CI 0.34–1.24, p 0.19).^[17]^ There was no difference in 28-day mortality.^[17]^ Kitagawa et al reported the low occurrence of *S argenteus* bacteremia, with 1.0% of all *S aureus* isolates causing bacteremia and 1.7% of MSSA isolates causing bacteremia being identified as *S argenteus*.^[18]^

S argenteus exhibits β-hemolysis and lacks golden pigment on blood agar. So far, the most feasible approach employed in clinic is based on the application of MALDI-TOF MS which can identify several specific signals that contribute to the resistance rate of *S argenteus* differentiation of bacterial species.^[5,15,16,19,20]^ Routine biochemical methods such as agglutination cannot distinguish *S argenteus* from *S aureus*.^[15]^ Up to date, there are no commercially available DNA-based assays for the identification of *S argenteus*.

The antibiotic is lower than that of *S aureus*.^[20]^ The penicillin-resistant isolates are common, while methicillin-resistant strains are rare. Some isolates are resistant to tetracyclines, aminoglycosides, clindamycin and/or erythromycin.^[21]^ In this case, the isolate is penicillin-resistant and sensitive to methicillin and other tested antibiotics, which is consistent with other reports.^[16,20,21]^ With the reports of methicillin-resistant *S argenteus* isolates were identified,^[3]^ the emergence of methicillin resistance in *S argenteus* shows the potential for obtainment of the methicillin resistance gene in its biological evolution. As the invasion and dissemination of *S argenteus* into healthcare facility environments with high antibiotic pressure, the threat of the emergence of methicillin resistance in *S argenteus* is possible and deserves further concern for public health benefits.

In this case, the *S argenteus* strain X17 was obtained from the blood culture on the first night of hospitalization. Early clinical studies suggested that *S argenteus* was probably associated with community-onset superficial skin lesions.^[22]^ The patient had long-term heavy drinking and smoking as well as chronic malnutrition, which may account for his immune deficiency. Studies show that immunocompromised patients may have increased risk of *S argenteus*.^[23]^ The patient probably developed septic shock after being infected by *S argenteus* in community. Because of the patient immune deficiency and the rapid progress of the community onset invasive infection, the patient died after a series of emergency treatment upon admission.

Semisynthetic penicillin, including nafcillin or oxacillin, are recommended as first-line antibiotics for serious MSSA infections, and cefazolin as an alternative.^[24]^ However, clinicians usually use empiric broad-spectrum beta-lactam antibiotics such as piperacillin/tazobactam for serious infections. Maya et al reported that 30-day mortality was significantly higher in patients with MSSA bloodstream infections treated with piperacillin/tazobactam compared with nafcillin, oxacillin or cefazolin.^[25]^ The increased mortality with piperacillin/tazobactam may due to the inability of tazobactam to induce staphylococcal β-lactamases. The staphylococcal β-lactamase is structurally a class A serine β-lactamase according to Bush classification. It is well known that a major target of β-lactamase inhibitors such as tazobactam is the class A serine β-lactamase of Gram-negative organisms, which is readily inhibited by these inhibitors. The β-lactamase producing isolates show elevated MICs beyond the susceptible range when inoculum of organisms is increased among BLBLIs including piperacillin/tazobactam.^[24]^ Both increased MICs and inoculum effect can lead to treatment failure. In our case, empirical antibiotic treatment with piperacillin/tazobactam was started at the beginning of admission, and the *S argenteus* strain X17 was positive for blaZ and resistant to benzylpenicillin. The patient died 3 days after admission. Although early mortality within 3 days in patients receiving piperacillin/tazobactam has not been studied, we speculate that the use of piperacillin/tazobactam may have contributed to the patient death, which should be considered in clinical practice.

## 4. Conclusion

*Staphylococcus* are common colonized bacteria in the oral cavity and the skin of healthy adult. It is reported that immunocompromised people are more vulnerable to infection by such bacterium and then develop bacteremia. The patient immune conditions appear to increase his susceptibility for *S argenteus* infection. The mortality of bloodstream infection caused by *S argenteus* is rather high, which should be paid attention in medical practice.

## Author contributions

**Conceptualization:** Yu Zhan.

**Formal analysis:** Yingmiao Zhang.

**Resources:** Xun Li, Yue Fan.

**Supervision:** Jing Wang, Hui Wang.

**Writing – original draft:** Yu Zhan.

**Writing – review & editing:** Ruizhi Tang, Zhongxin Lu.
